# Results of Studying the correlation Between Stress and Depression of Miner Workers

**DOI:** 10.1192/j.eurpsy.2025.1364

**Published:** 2025-08-26

**Authors:** P. Sarantuya, B. Purev, M. Tuul, T. Urtnasan

**Affiliations:** 1 Medical Department; 2 Basic Medical Department, Etugen University, Ulaanbaatar, Mongolia

## Abstract

**Introduction:**

Mining work and environment are the hardest and most difficult jobs in the labor market, increasing the risk of physical and mental illness. A study was conducted to determine the level of stress in mine workers and evaluate the relationship between depression and stress.

**Objectives:**

To determine the level of stress in mine workers and evaluate the relationship between depression and stress.

**Methods:**

Questionnaires were collected from mine workers using the stress detection test (SRQ) and depression detection test (PHQ9) developed by the World Health Organization for doctors.

**Results:**

2.2% (95) of the respondents are male and 7.8% (8) are female workers. Of these, 70.9% work night shifts, 24.3% do not work night shifts, and 4.8% occasionally work night shifts. Comparing the stress levels of the respondents by age, 1.9% (2) of 20-24-year-olds are moderately stressed, 3.9% (4) of 25-29-year-olds are moderately stressed, and 4.8% (5) are stressed. 30-34-year-olds have moderate stress, 1.9% (2) have high stress, 1% (1) of 35-39-year-olds have moderate stress, 1.9% (2) have high stress, and 1% (1) have high stress. 40-44 have moderate, 1% (1) high, and 1% (1) 45-49 have moderate stress. The depression status of the participants compared to the 20-24-year-olds was 1% (1) transient mood depression, 1% (1) moderate depression, and 25-29-year-olds self-correcting transient mood 4.8% depression (5), 4.8% moderate depression (5), 1% (1) moderate depression, 1% (1) severe depression, 5.8% (6) depressed, 4.8% ((5)) moderate depression, 1.9% (2) moderate depression, 35-39-year-old spontaneous self-correction (68%). 7) with depression, 1.9% (2) with moderate depression, temporary self-correction at the age of 40-44 1.9% (2) with depression, 1% (1) with severe depression, temporary at the age of 45-49 2.9% (3) have depression. According to this, it can be considered that 25-44-year-olds are at risk of depression with a high depression index.

**Conclusions:**

The study concluded that 25-44-year-olds are at risk of depression with a high depression index, indicating that they are at risk of depression.

**Disclosure of Interest:**

None Declared

